# Case report of invasive ductal carcinoma of the breast in a Pakistani male aged 55

**DOI:** 10.1007/s12672-026-04996-0

**Published:** 2026-04-10

**Authors:** Shahid Aziz, Faisal Rasheed, Samra Bibi, Adil Shahzad, Sidra Riaz, Haseeb Noor, Umer Zeeshan Ijaz, Simone König

**Affiliations:** 1 Institute of Allied Health Sciences, Wah Medical College, National University of Medical Sciences, Rawalpindi, Pakistan; 2 Patients Diagnostic Lab, Pakistan Institute of Nuclear Science and Technology, Islamabad, Pakistan; 3 Institute of Nursing, Wah Medical College, Wah Cantt, Pakistan; 4 Fazaia College of Nursing and Allied Health Sciences, Air University, Islamabad, Pakistan; 5 Policy and Coordination Wing, Ministry of Science and Technology, Islamabad, Pakistan; 6 Department of Gastroenterology, Farooq Hospital, Akhtar Saeed Medical College, Rawalpindi, Pakistan; 7 Advanced Research Centre, Infrastructure and Environment, James Watt School of Engineering, University of Glasgow, 11 Chapel Lane, Glasgow, G11 6EW UK; 8 IZKF Core Unit Proteomics, University of Münster, Münster, Germany

**Keywords:** Breast cancer, Male, Case report, Clinical features, Incisional biopsies, Histopathology, Invasive ductal carcinoma

## Abstract

**Background:**

The male breast cancer is a rare malignancy comprising for less than 1% of all breast cancers. The late and incorrect diagnosis of this cancer causes increased incidence rates of metastatic spread within patients. In Pakistan, the male breast cancer is frequently diagnosed at an average age of 55 to 58 years. Additionally, different risk factors including old age, hormonal imbalances, as well as family history showed association with this cancer. Moreover, recent literature showed that the neoadjuvant chemotherapy can result in pathologic complete responses in male breast cancer patients, yet its use in real-world practice is low, highlighting the gap in clinical implementation. Therefore, we present a clinical case report of male breast cancer to identify the critical importance of considering this malignancy in young males with breast masses and to discuss the unique challenges in their management.

**Case presentation:**

We present the case of a 55-year-old normotensive and normoglycemic male office clerk with a four-months history of mild pain and lump in the left breast. He did not have a family history of breast cancer. Physical examination revealed a hard lump with overlying skin changes suggestive of malignancy. Incisional biopsies confirmed invasive ductal carcinoma, grade II with ER-positive, PR-negative and HER2/neu-negative status with no lymphovascular invasion. Imaging studies showed lobulated mass in the left breast with axillary lymphadenopathy in the ipsilateral axilla, renal calculus and benign lytic lesions. The patient was initially treated with planned neoadjuvant chemotherapy including four cycles of doxorubicin and cyclophosphamide scheduled over twelve weeks. However, a follow-up biopsy performed after nine weeks showed persistent invasive carcinoma. Consequently, the treatment plan was reevaluated subsequently. Because of the tumor’s ER-positive status and suboptimal response to neoadjuvant chemotherapy, the treatment plan was changed to endocrine therapy such as tamoxifen (10 mg twice daily). The patient was clinically stable during discharge and follow-up was advised.

**Conclusion:**

This case report of male breast cancer highlights the unique aspects and challenges of diagnosing and treating cancer emphasizing the importance of early diagnosis, hormone receptor status for treatment planning and ongoing research to manage cancer effectively. Moreover, it also highlights the necessity of imaging in such cases as clinical examination may fail to pick significant findings. Similarly, absence of lymphovascular invasion on biopsy is not always associated with absent lymph node involvement on imaging. Moreover, this case report also demonstrated the standard diagnostic and therapeutic pathways for a typical case of ER-positive invasive ductal carcinoma in a male patient. This clinical case report highlights the crucial in recognizing chemotherapy-resistant disease and supporting the clinicians in the timely initiation of the endocrine therapy.

## Background

Male breast cancer, though infrequent, presents distinctive characteristics compared to its more common occurrence in females. Statistics indicate that it comprises less than 1% of all male cancers [1] and approximately 1% of all breast cancers [2] with an estimated incidence rate of less than 1 per 100,000 men, contributing to 0.1% of cancer-related deaths in males [3, 4]. On average, male breast cancer manifests between the ages of 60 and 70 [5], with a mean age of 67 [6], suggesting a slightly older age at diagnosis compared to women [7]. Male breast cancer is more common in older than younger men with a higher probability of its spread to axillary lymph nodes [8]. Most of the symptomatic patients diagnosed with breast cancer have a low survival rate [9]. Estrogen receptor-α (ER), progesterone receptor (PR), HER2, androgen receptor, and BRCA2 represent extensively studied biomarkers for this purpose [10].

Invasive ductal carcinoma (92.6%) is the most prevalent subtype of male breast cancer as compared to lobular carcinoma and among those patients, HER2 positivity was 21.6% and ER and PR positivity was 96.4% and 71.4%, respectively [11]. There are some challenges in diagnosis of male breast cancer including lack of public awareness which leads to stigmatization as well as late presentation, absence of routine screening, resulting in advanced staged cancer [12] Moreover, during staging, the axillary lymph node assessment is critical yet difficult in male breast cancer because of higher frequency of nodal involvement during diagnosis.

Nodal status is considered strongest sign for prognosis which directly indicates the adjuvant therapy decisions for both of systemic treatment as well as radiation [13]. Although, clinical and ultrasonographic assessment of axilla is mostly inaccurate, as nodes possess micro-metastases which may appear normal, leading to the dangerous disparity between pre-surgical imaging and final diagnosis which emphasis the crucial role of sentinel lymph node biopsy specimen for ultimate staging and adequate treatment [14].

Here we present the case of a 55-year-old male with a four-month history of mild pain and a lump in the left breast.

## Case description

The 55-years old male office clerk, who was normotensive and normoglycemic with no other co-morbidity and no history of breast cancer in his family, presented to his primary health care practitioner at a tertiary care hospital with mild pain and a lump in the left breast for four months. On physical examination, the physician detected a hard lump having a size of 2 × 1.5 cm in the upper quadrant of the left breast. The lump was fixed to the underlying structure; the overlaying skin was red, edematous, and darkly ulcerated. Moreover, no palpable bilateral axillary lymph nodes or lumps in the contralateral breast were found during examination. Because of subclinical nodal involvement, patient was advised CT scan for early detection of deep lymph node disease.

The patient was admitted with blood pressure (132/102) mmHg, (heart rate) 75 per min and SpO_2_ 99% to rule out malignancy. Only incisional biopsies were taken under local anesthesia for histopathological examination. Moreover, no lymph nodes were excised or biopsies taken before neoadjuvant chemotherapy. The incisional biopsies were preferred over core needle biopsies due to tumor’s ulceration as well as to ensure sufficient viable tissue for final diagnosis and biomarkers identification. The patient was vitally stable (blood pressure 108/74 mmHg, heart beats 83 per min, SpO_2_ 98%) post biopsy and discharged with prescribed treatment including Co-Amoxiclav BD and Paracetamol and orphenadrine combination for five days followed by Pregabalin 75 mg BD for 7 days and wound dressing change on alternative days. The patient was advised for follow-up with histopathology reports.

**Fig. 1:**
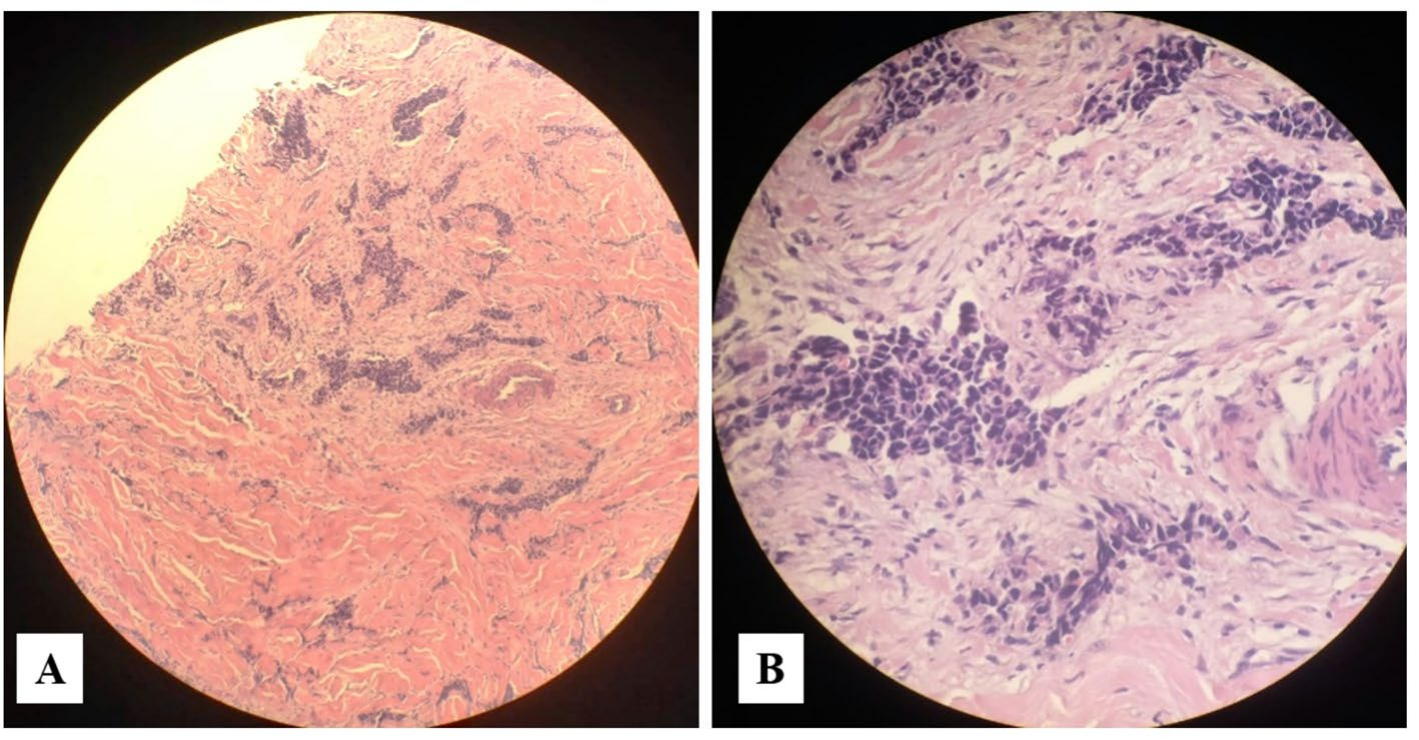
Histopathological Examination Revealed Invasive Ductal Carcinoma. The section showed irregular nests and cords of malignant epithelial cells within a dense fibrous stroma, with a desmoplastic reaction as well as mild chronic inflammation (**A**; H&E, 40×). While at higher magnification (**B**; H&E, 400×), the tumor cells showed nuclear pleomorphism, hyperchromasia, prominent nucleoli, and a high nuclear/cytoplasmic ratio, consistent with invasive ductal carcinoma

The biopsy specimens appeared under the microscope as gray brown tissues with fat collectively measuring 1.3 × 1 × 0.5 cm. The histopathological examination described them as invasive ductal carcinoma of histological and nuclear grade II with no lymphovascular invasion (Fig. 1).

The patient was advised for further investigations including complete blood picture, liver and renal functions tests, immunohistochemical examinations (ER, PR, HER2/neu), two-dimensional echocardiogram **(**2D echo) and bone scan as well as computed tomography (CT) scan of chest, abdomen and pelvis.

### Pre-treatment blood tests

The patient’s blood tests revealed macrocytosis characterized by raised mean corpuscular volume (MCV), with normal levels of hemoglobin (Table 1).

**Table 1:** Patients Blood Tests Result Pre- and Post-Neoadjuvant Chemotherapy: Summary of patient’s blood picture as well as liver and renal function tests pre-/post-anti-cancer treatment along with corresponding reference ranges for each parameter

Complete blood picture	Results		Units	Reference ranges
	Pre-treatment	Post-treatment		
HB	13.6	10.9	g/dL	13.0-16.5
HCT	40.9	34	%	40–52
RBC	3.88	3.5	×10E6/µl	4.5-6.0
MCV	105.4	98	Fl	80–100
MCH	35.1	32	Pg	27–34
MCHC	33.3	32	g%	30–35
WBC	6.5	3.9	×10e3/µl	4.0–11.0
Neutrophils%	53	65	%	40–75
Lymphocytes%	39	17	%	20–45
Monocytes	6	14	%	2–10
Eosinophils%	2	3	%	1–6
Basophils%	0	1	%	0–1
Platelets	165	268	×10e3/µl	150–400
Liver function test				
S. Total Bilirubin	0.5	0.34	mg/dl	0.1-1.0
ALT	55	55	U/L	< 40
AP	169	111	U/l	39–117
Renal function test				
Urea	35	26	mg/dl	17–49
Creatinine	1.0	0.82	mg/dl	0.4–1.3

### Immunohistochemical analysis

**Fig. 2:**
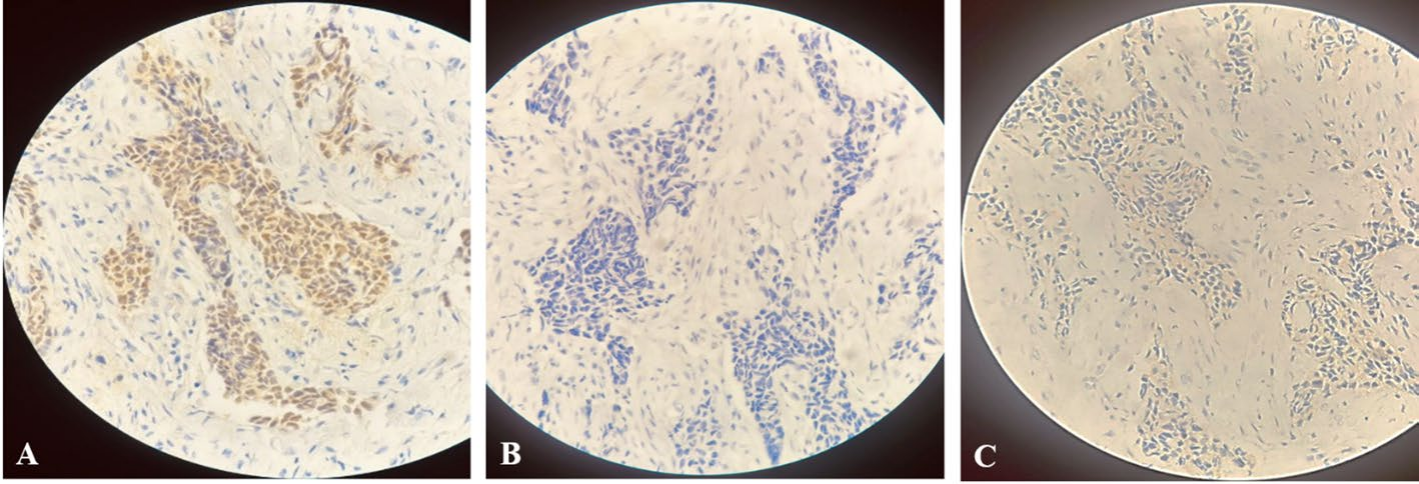
Immunohistochemical Analysis of Male Breast Cancer Biopsies. The biopsies showed strong nuclear positivity for ER (**A**: 10×), while PR (**B**: 10×) and HER2 (**C**: 10×) expression are negative

Immunohistochemical examination revealed ER-positivity (proportion of nuclei stained: 45%, proportion score: 4, intensity score: 3, total 7/8), PR-negativity (proportion of nuclei stained: 0%, proportion score: 0, intensity score: 0, total 0/8), and HER2/neu-negativity (membrane staining score 0), (Fig. 2).

### 2D Echo

**Fig. 3:**
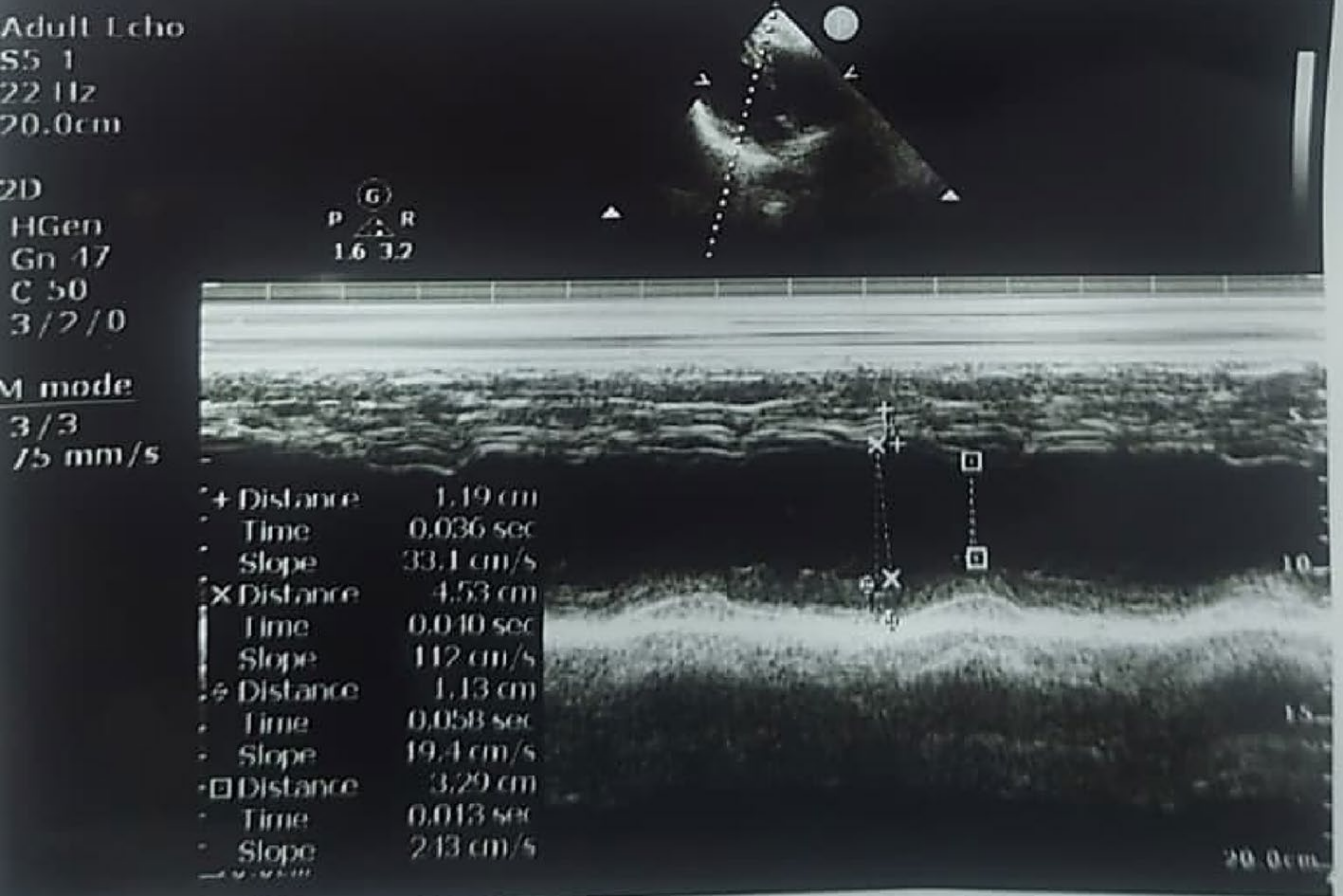
2D Echocardiogram of Male Breast Cancer Patient. The 2D Echocardiogram showed normal cardiac structure with normal ejection fraction

The echocardiogram of the patient found regular features including normal sized cardiac chambers, preserved left ventricular systolic function, no regional wall motion abnormality, normal in structure and function appearance of valve and right side of heart, grade I diastolic dysfunction (E/A reversal); no clots or pericardial effusion were seen (Fig. 3), (Table 2).

**Table 2:** Summary of 2D Echo Findings: Assessment of cardiac health and function

Echo measurements	Normal values (MM)	Patient results
LV end diastolic	35–55	45
LV end systolic	25–41	36
Septal thickness end diast	12	10
Left atrium	19–40	32
Aortic root	20–37	30
Ejection fraction	54–75%	60%

## Radiological assessment

### CT chest, abdomen and pelvis

CT scans revealed a significant lobulated, enhancing mass lesion in the outer half of the left breast, measuring 4.2 × 1.7 × 3.8 cm. This lesion, with spiculated margins, abutted the pectoralis muscle posteriorly and involved the overlying skin, causing thickening and retraction. Moderate soft tissue stranding was noted in the surrounding subcutaneous fat. Multiple enlarged axillary lymph nodes (level I and II) on the left side, measuring 8.9 mm in short axis with loss of fatty hilum, were observed in the ipsilateral axilla, while the right breast appeared normal with a few lymph nodes in the right axilla maintaining intact fatty hila. No suspicious lung nodules, significant mediastinal, or hilar lymphadenopathy were present. The cardiovascular structures, including the aorta and pulmonary vasculature, appeared normal with no pericardial or pleural effusion detected.

In the abdomen, the liver was normal in size and texture with a small calcified granuloma in segment VI, and the gallbladder showed no abnormalities. The pancreas and spleen were of normal size and texture, and no focal lesions were identified. The kidneys were normal in size, shape, and texture, though a large calculus was noted at the lower pole of the left kidney, measuring 1.1 × 1.6 cm, with associated perinephric fat stranding. There was no evidence of hydronephrosis, cysts, or masses in the kidneys, and the adrenal glands were normal. The gut loops and ileocecal junction appeared normal with no signs of wall thickening or dilatation, and the urinary bladder and prostate gland were also normal. Degenerative changes were seen in the visualized spine, with two tiny lytic areas in the right 4th and 5th ribs and a lytic lesion in the right ilium at the SI joint, all with well-defined sclerotic margins.

In conclusion, the CT scan revealed a left breast mass involving the overlying skin and underlying muscle with left axillary lymphadenopathy, classified as T4N1/2Mx. A left renal calculus was also noted. Additionally, tiny lytic lesions with well-defined sclerotic rims were observed in the right 4th and 5th ribs, likely benign; however, considering the known malignancy, a bone scan was suggested for further evaluation. No hepatic or lung metastases were found.

### Bone scan

**Fig. 4:**
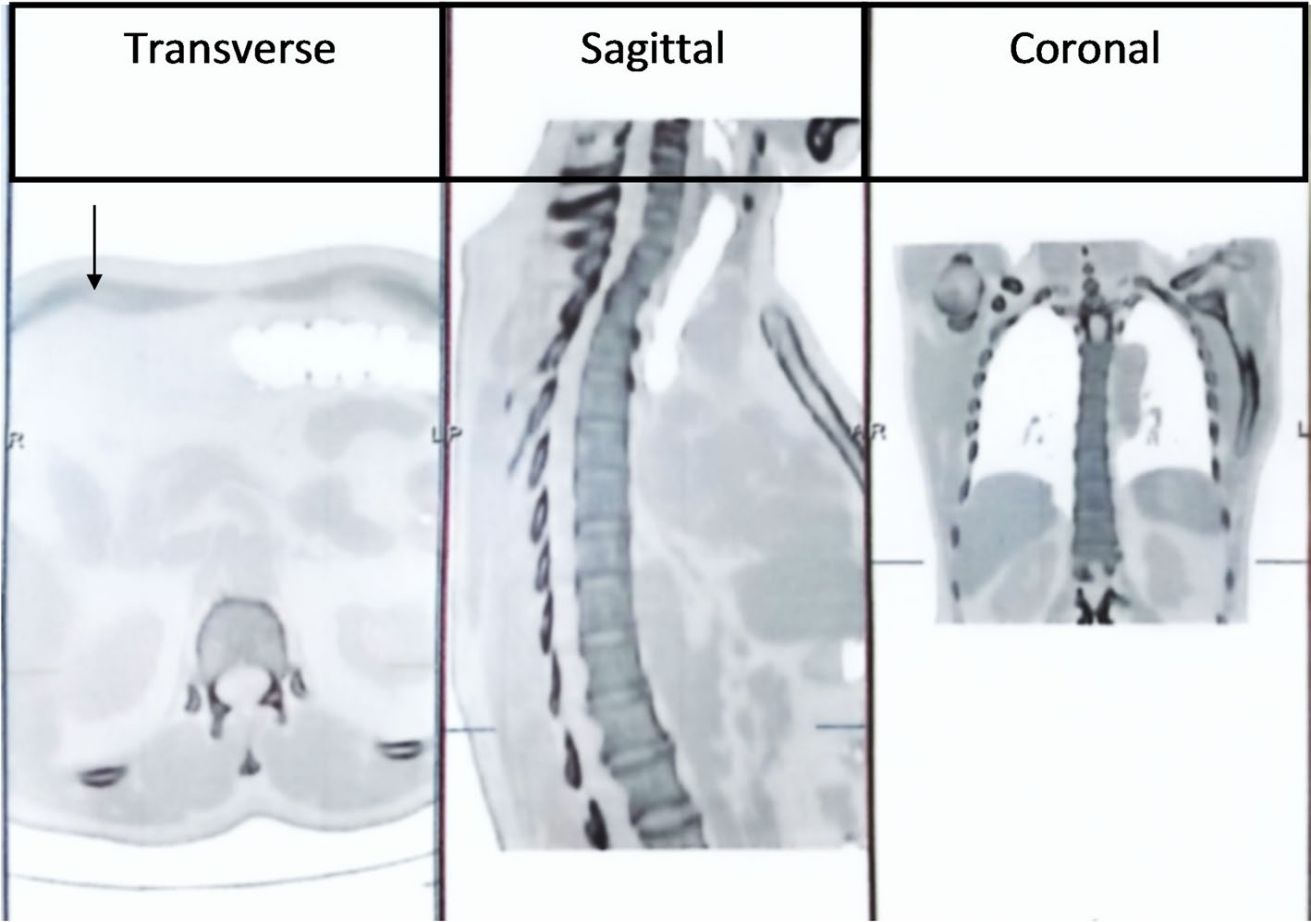
SPECT/CT Findings of Chest. **A** Transverse, **B** Sagittal, **C** Coronal slices of SPECT/CT images (arrow) of male breast cancer patient showing incidental lytic lesions in right rib

**Fig. 5:**
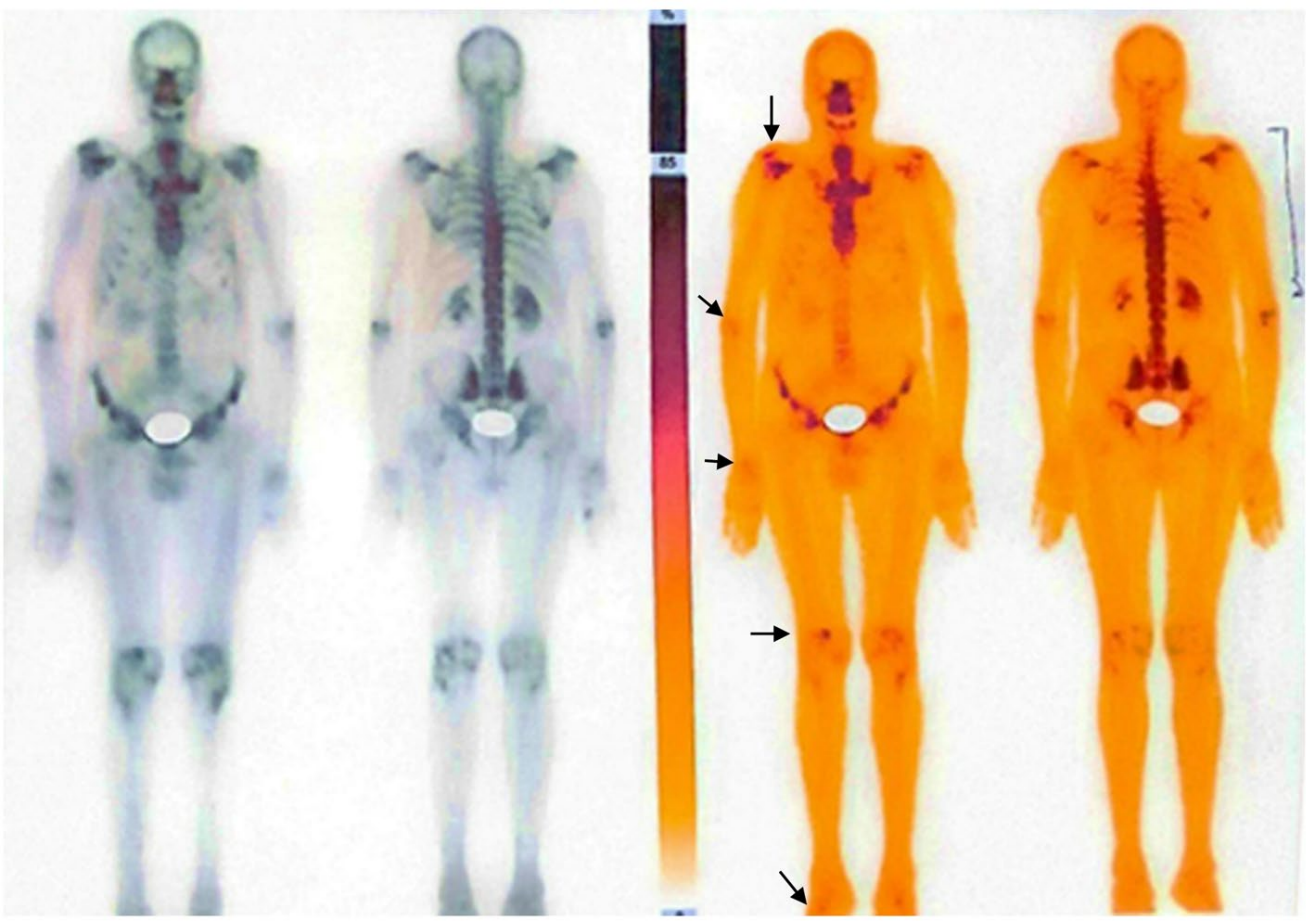
Whole Body Bone Scan with Tc-99 m MDP: Whole-body bone scan acquired 2.5 h post-injection of 20.0 mCi of Tc-99 m MDP, with static images obtained in both anterior and posterior projections. The scans revealed multifocal degenerative changes (arrows) in the shoulders, elbows, wrists, knees, ankles The rest of the skeleton showed bilaterally symmetrical and normal tracer distribution. Additionally, the scan was negative for skeletal metastasis

The whole body was scanned with 20.0 mCi of Tc-99 m MDP administered intravenously. The static images were acquired in both anterior and posterior projections at 2.5 h post-injection followed by SPECT/CT of chest (Fig. 4). The multifocal degenerative changes in patient body parts including shoulders, elbow, wrist, knee, ankle joints as well as dorsolumbar spin were observed during whole-body scans. Furthermore, the remaining parts of skeleton showed bilaterally symmetrical as well as normal tracer distribution (Fig. 5). The scan showed no evidence of skeleton metastasis.

### Treatment

**Fig. 6:**
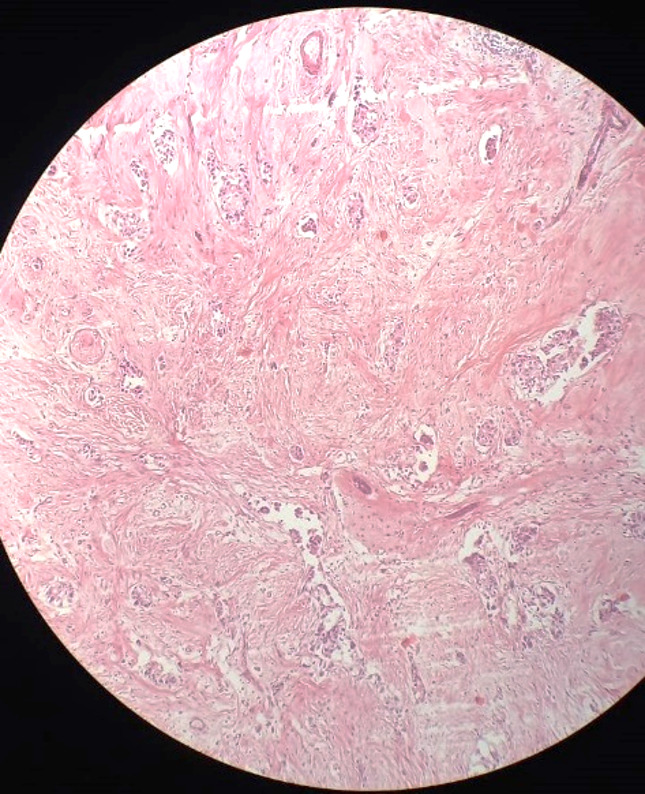
Post-treatment Histopathological Examination of MaleBreast Biopsies. The micrographic section (H & E: 400×) showed foci of residual invasive ductal carcinoma in male breast cancer patient

Initially, the patient was treated with planned neoadjuvant chemotherapy consisting of 4 cycles of doxorubicin (110 mg) and cyclophosphamide (1100 mg) scheduled over 12 weeks. Following the initial neoadjuvant chemotherapy, the planned follow-up biopsy after nine weeks revealed persistent invasive carcinoma (Fig. 6) indicated the suboptimal response to chemotherapy. The planned mastectomy was omitted after chemotherapy failure. Consequently, the treatment plan was reevaluated for timely disease management.

In light of the tumor’s ER-positive status and residual disease, the treatment plan was modified to endocrine therapy with tamoxifen (10 mg twice a day) in order to improve the outcomes as well as avoid unnecessary toxicity even after persistent residual disease after initial treatment. Subsequent investigations (Table 1) indicated disease stability, and the patient was advised to continue regular follow-up.

After treatment, there have been notable changes in the patient’s health markers. The treatment has led to a decrease in hemoglobin, hematocrit, and RBC count, which indicated that the patient was experiencing mild anemia, a common myelosuppressive side effect of the chemotherapy regimen.

Additionally, the white blood cell count had dropped due to the treatment’s impact on bone marrow. The platelet count had increased possibly also as a response to the treatment. Kidney function had improved, with both urea and creatinine levels decreasing and staying within the normal range. These results highlighted the need for ongoing monitoring anemia and white cell counts (Table 1).

## Discussion

This case report of 55-year-old male diagnosed with breast cancer emphasize various critical diagnostic as well as therapeutic challenges. The suboptimal response of patient to neoadjuvant chemotherapy, followed by successful switch to endocrine therapy, strongly strengthens the importance of hormone receptor status to direct treatment decisions for breast cancer in male patients in accordance with established guidelines [15]. The neoadjuvant chemotherapy is used in advanced stage male breast cancer for early treatment of micro-metastatic disease, to downstage the tumor as well as improvement of surgical options [16]. Although, mostly the breast cancers in male are ER-positive which show suboptimal responses to anti-cancer therapies [17]. The presence of residual carcinoma after treatment with neoadjuvant chemotherapy indicates the chemoresistance and emphasize the endocrine-driven biology of tumor. Selection of tamoxifen is suitable for targeting ER signaling, reduction of recurrence risk and especially provision of non–cross-resistant systemic anti-cancer therapy [18]. In present case, patient personalized treatment approach was used in which patient was initially treated with neoadjuvant therapy followed by endocrine therapy to optimize long-term outcomes.

Moreover, in present case report, the patient had lymphadenopathy diagnosed during radiological imaging in spite of absence of lymphovascular invasion during histopathological examinations of biopsies which point towards the evaluative clinical lesson that radiographic staging is essential and histopathological findings alone cannot suffice. Additionally, the age of the patient is especially younger as compared to the reported global mean, that coincide with domestic data indicating an earlier onset in Asian populations [19].

Around 42% of male breast cancer cases are diagnosed at advanced stages III or IV [20] likely due to the fact that males do not seek medical care for breast lumps as quickly as women do. Most men are unaware that they also can develop breast cancer which may lead to delay to recognize signs and symptoms associated with this cancer. Furthermore, male breast cancer is still stigmatized as feminine disease-causing shame and embarrassment in some men upon diagnosis. In some communities, confessing to suffering from any ailment linked to femininity can bring about ridicule from the rest of the society hence leading to acts of ostracism. This discourages men from approaching health facilities for medical attention when they fall sick.

Another possible reason could be that the tumor grows closer to the skin in males, which increases the probability of infiltration into the middle layer of the skin such as the dermis [21] which was also seen in this case. The men diagnosed with breast cancer have poor prognosis, particularly at younger age, when it may be misdiagnosed due to gynecomastia [22]. Breast cancer in men is mostly diagnosed at an age of ~ 65 years [23]. In the present case, the patient was diagnosed at the age of 55 years and his treatment started approximately after two months. A research study by Hanna et al. (2020) indicated that a delay of four weeks in cancer treatment correlates with an increased mortality rate. Hence, early detection is imperative for the timely management of this cancer.

Family history is a major risk factor for breast cancer [24] as are some genetic diseases including Klinefelter’s syndrome and Cowden’s disease [25]. In our case, the patient did not have a family history of breast cancer and he was not tested for genetic diseases due to financial constraints.

Our case report has various limitations. Primarily, this is a single clinical case report. Therefore, the findings of this case are descriptive which can’t be generalized. Another significant limitation of this case report is the lack of genetic testing in male [26]. To overcome this limitation, all men should be offered genetic counseling as well as testing for germline mutations (especially BRCA2 genes), as the frequency of pathogenic variants is higher as compared to female breast cancer [27].

There is no evidence that all men diagnosed with breast cancer need radiological assessment. However, it is important to examine the contralateral breast to look for suspicious lesion. Moreover, male patients who survive after breast cancer, have a higher tendency of developing secondary cancer [28]. The risk of cancer in the contralateral breast is as much as two to four times higher in men as compared to females [29]. In this case report, the CT scan was opted as initial cross-sectional imaging modality. it provided a single, effective modality to assess both the breast, axilla, as well as other unexpected clinical findings, assisting clinical decision-making for this patient of breast cancer.

Males have significantly less mammary parenchyma than women. Clinical correlation of blood tests results, radiological findings and histopathological analysis of percutaneous biopsies must be carried out for differential diagnosis [30]. In this case, before treatment the patient was diagnosed with macrocytosis because of higher MCV possibly due to nutritional deficiencies or early bone marrow stress. After treatment, the anemia worsened leading to leukopenia which suggested chemotherapy-induced bone marrow suppression. Moreover, liver enzymes also mildly raised indicating hepatic involvement, which can also cause macrocytosis.

For effective treatment, core needle biopsy is required to diagnose invasive breast cancer and to evaluate ER, PR and HER-2 status [31]. Receptors such as ER and PR play a significant role in male breast cancer. These receptors are expressed in 91% (ER) and 80% (PR) of male breast cancer cases [32]; in our case the cancer was ER-positive. Moreover, invasive ductal carcinoma is more frequent in male breast cancer with ER and PR positivity and shows unusual characteristics [33].

HER-2 overexpression is linked to the worst prognosis for breast cancer patients [34]. The expression levels of these receptors in both male and female play vital role in the treatment selection for male breast cancer [35]. Recent studies revealed that ER and PR show sex-specific binding and that males have more expression of ER than females [36]. Furthermore, male breast cancer most commonly spreads to bones [9].

The treatment plans for male breast cancer are mostly inferred from data in women breast cancer [37]. Parameters such as tumor size, expression of ER, PR and HER-2 and age-related co-morbidities must be considered [38]. Men diagnosed with breast cancer often present risk factors, such as chronic hepatopathies, which are directly related with neoplasm [39]. Some other factors including increased body mass index and abdominal obesity [40] and infertility [41] may also be associated to breast cancer. Given the relatively smaller size of male mammary parenchyma, the preferred surgical intervention typically involves modified radical mastectomy. In our case, the surgeons removed only the abnormal area through incisional biopsies rather than whole breast.

The patient was treated first with Doxorubicin and Cyclophosphamide followed by Tamoxifen in a patient centered approach. As a result of ER-positive expression, endocrine interventions are needed with such anti-cancer drugs, but some male patients diagnosed with breast cancer do not tolerate Tamoxifen because of its side effects [42]. Tamoxifen is still considered as an optimal adjuvant therapy for male breast cancer along with endocrine responsive diseases. Knowledge on the impact of adjuvant chemotherapy regarding both rate and overall survival is not extensively explored [43]. Several studies have shown enhanced disease-free as well as overall survival when compared to historical controls through the use of adjuvant anthracycline-based therapies [44].

## Conclusions

We discuss a case of breast cancer in a 55-year-old male. It underscores the importance of vigilance towards male breast pathology, particularly in the context of atypical presentations. The diagnostic journey, from clinical examination to histopathological confirmation and subsequent imaging, highlights the multidisciplinary approach necessary for accurate assessment and management. It also highlights the limitations of clinical examination to detect axillary lymph nodes. This case report showed that radiological imaging i.e., CT and histopathological analysis provide non-redundant and complementary information. The CT scan found suspicious lymph nodes which were not predicted by the Lymphovascular Invasion-negative biopsy, highlighting the crucial role of cross-sectional imaging to identify comprehensive staging, especially when histopathological findings of biopsy specimen appear deceptively indolent. Additionally, it also emphasizes how the selection of imaging modality including axillary ultrasound versus CT scan may differ depending on local expertise. Moreover, the health care authorities should also address the limitation of genetic testing in both genders to create the awareness to halt the spread of this cancer in communities around the globe especially in Pakistan.The rarity of male breast carcinoma, coupled with its potential for advanced disease at presentation, requires a thorough evaluation to guide personalized treatment strategies. Despite its low incidence and mortality rates in comparison to female breast cancer, understanding the unique aspects of male breast cancer remains crucial for optimizing diagnostic and therapeutic approaches. Further investigation into age-related differences in diagnosis and potential sex-specific nuances in disease progression is warranted to enhance patient outcomes and support for this understudied population. More research is needed to elucidate optimal management strategies and improve outcomes for individuals affected by this rare malignancy.

## Data Availability

The data are available from the authors upon request.

## Abbreviations

BC: Breast cancer
IDC: Invasive ductal carcinoma
ILC: Invasive lobular carcinoma
ER: Estrogen receptor
PR: Progesterone receptor
CT: Computer tomography
LFTs: Liver function tests
RFTs: Renal function tests
Hb: Hemoglobin
WBC: White blood cell
ALT: Alanine transaminase
AST: Aspartate transaminase
ALP: Alkaline phosphatase
RBC: Red blood cell
MCV: Mean corpuscular volume
